# 1251. Strengthening Antimicrobial Stewardship Programs through Public Health Support

**DOI:** 10.1093/ofid/ofad500.1091

**Published:** 2023-11-27

**Authors:** Jane Kriengkauykiat, Tisha Mitsunaga, Erin Epson

**Affiliations:** HAI Program, California Department of Public Health, Richmond, California; California Department of Public Health, Richmond, California; California Department of Public Health, Richmond, California

## Abstract

**Background:**

Antimicrobial stewardship programs (ASP) improve patient outcomes and reduce resistance and *Clostridioides difficile* infections. The California Department of Public Health (CDPH) Healthcare-Associated Infections (HAI) Program developed an Honor Roll to recognize and promote ASP and offers consultation service to hospitals seeking to strengthen their ASP. We evaluated the service and compared ASP who sought consultation (consult) versus those who did not (non-consult).

**Methods:**

We included ASP applying for Honor Roll between Sep 2020 to Mar 2022. Each hospital ASP completed a standardized application and provided documentation for Bronze, Silver, or Gold designations. Criteria increases as designation increases; details are on the Honor Roll website (www.cdph.ca.gov/Programs/CHCQ/HAI/Pages/Honor_Roll.aspx). ASP may apply for upgrade within one year of designation. Consultation was initial review of ASP; mentorship was ongoing support beyond initial review. We reviewed consultation and mentorship meetings for themes in type(s) of support provided. For ASP that applied for upgrades, we compared consult to non-consult, including facility characteristics, and receipt of upgrade.

**Results:**

There were 168 ASP (176 hospitals) Honor Roll enrollees (44% of California hospitals): 29 consults with 13 receiving mentorship, and 139 non-consult. Community hospital was the majority facility type which was similar between consult, 16/29 (55%), and non-consults, 80/139 (58%). The proportion of community hospitals ≤250 beds was higher for consult 13/29 (45%) vs non-consult 52/139 (37%). Thirty ASP applied for upgrades: 13/13 (100%) consults received upgrades vs 10/17 (59%) non-consults. Among non-consults, 4 received no upgrades and 3 received a lower designation (i.e., applied for Gold, received Silver). Among consults, 15/29 (52%) required support in strengthening their program, 10/29 (30%) application assistance, and 4/29 (14%) metrics or tracking. Approximately 7% of both consults and non-consults were in lower population- dense areas (< 1000 population/square mile).

**Conclusion:**

Public health can support facilities improve their ASP through technical assistance and consultation; ASP in smaller facilities might require additional support.

**Disclosures:**

**All Authors**: No reported disclosures

